# Physiological-induced conductive response evaluation in specific muscle compartments under hybrid of electrical muscle stimulation and voluntary resistance training by electrical impedance tomography

**DOI:** 10.3389/fphys.2023.1185958

**Published:** 2023-07-18

**Authors:** Bo Sun, Panji Nursetia Darma, Prima Asmara Sejati, Tomoyuki Shirai, Kosei Narita, Masahiro Takei

**Affiliations:** ^1^ School of Mechanical and Precision Instrument Engineering, Xi’an University of Technology, Xi’an, China; ^2^ Department of Mechanical Engineering, Graduate School of Science and Engineering, Chiba University, Chiba, Japan; ^3^ Department of Electrical Engineering and Informatics, Vocational College, Universitas Gadjah Mada, Yogyakarta, Indonesia; ^4^ MTG Co., Ltd., Nagoya, Japan

**Keywords:** electrical impedance tomography (EIT), electrical muscle stimulation (EMS), voluntary resistance training, *hybrid*EMS, physiological-induced conductive response

## Abstract

**Objective:** The physiological-induced conductive response has been visualised for evaluation in specific muscle compartments under hybrid (*hybrid*EMS) of electrical muscle stimulation (EMS) and voluntary resistance training (VRT) by electrical impedance tomography (EIT).

**Methods:** In the experiments, tendency of conductivity distribution images **σ** over time was clearly detected for three specific muscle compartments, which are called *AM*_1_ compartment composed of biceps brachii muscle, *AM*_2_ compartment composed of triceps brachii muscle, and *AM*_3_ compartment composed of brachialis muscle, under three training modalities.

**Results:** From the experimental results, the tendency of physiological-induced conductive response are increased in all three training modalities with increasing training time. Correspondingly, the spatial-mean conductivity <**σ**>_*AM*1,*AM*2,*AM*3_ increased with the conductance value *G* and extracellular water ratio *β* of right arm by bio-impedance analysis (BIA) method. In addition, *hybrid*EMS has the greatest effect on physiological-induced conductive response in *AM*_1_, *AM*_2_, and *AM*_3_. Under *hybrid*EMS, the spatial-mean conductivity increased from <**σ** ^*pre*^ > _*AM*1_ = 0.154 to <**σ** ^23mins^ > _*AM*1_ = 0.810 in *AM*_1_ muscle compartment (*n* = 8, *p* < 0.001); <**σ** ^*pre*^ > _*AM*2_ = 0.040 to <**σ** ^23mins^ > _*AM*2_ = 0.254 in *AM*_2_ muscle compartment (*n* = 8, *p* < 0.05); <**σ** ^*pre*^ > _*AM*3_ = 0.078 to <**σ** ^23mins^ > _*AM*3_ = 0.497 in *AM*_3_ muscle compartment (*n* = 8, *p* < 0.05).

**Conclusion:** The paired-samples *t*-test results of <**σ**>_*AM*1,*AM*2,*AM*3_ under all three training modalities suggest *hybrid*EMS has the most efficient elicitation on physiological induced conductive response compared to VRT and EMS. The effect of EMS on deep muscle compartment (*AM*_3_) is slower compared to VRT and *hybrid*EMS, with a significant difference after 15 min of training.

## 1 Introduction

Voluntary muscle strength is improved by expanding muscle mass in specific muscle compartments (Herbert et al., 1998). For instance, even lifting weights for as little as three seconds a day results in a positive impact on muscle strength (Sato et al., 2022). Regular muscle training for both young and elders enables the prevention of skeletal muscle disorders (Pennings et al., 2011), such as muscular dystrophy (Ansved, 2001), neuromuscular conditions (Pareja-Blanco et al., 2014), sarcopenia (Cheng et al., 2022). Resistance training is not a challenging task for people who have a training habit regularly, but not for the common people who never practice in their daily life. Furthermore, the recovery period is also difficult for people who suffer from prolonged immobility caused by bed rest. Hence, another type of training known as electrical muscle stimulation (EMS) has become a straightforward solution as a form of exercise and mobilization that does not require active participation with the capability to be applied to immobilized people (Karatzanos et al., 2012). Unfortunately, EMS training is often considered to yield a biased result (Fleckenstein et al., 1988). Effective resistance training requires professional instruction and subjects need to perform standard movements in order to achieve the desired training results (Fisher et al., 2022). In this regard, to improve the voluntary muscle strength effectively with a lower effort, hybrid of electrical muscle stimulation and voluntary resistance training called *hybrid*EMS was proposed (Deley et al., 2011). *hybrid*EMS offers optimum training with beneficial effects on muscle strength (Deley et al., 2011). However, the lack of a direct observation tool for physiological response before and after *hybrid*EMS training has created a great need for real-time muscle compartments evaluation.

Conventionally, two methods are usually considered to evaluate muscle mass. On one hand, conventional tomographic methods such as magnetic resonance imaging (MRI) (Dahlqvist et al., 2020) and dual-energy X-ray absorptiometry (DXA) (Andreoli et al., 2009) are currently recommended for evaluating muscle mass. MRI is based on the principle of nuclear magnetic resonance, which is dependent on the different attenuation of the energy released in different structural environments within matter. The location and type of atomic nuclei that make up the object are detected by applying a gradient magnetic field to the emitted electromagnetic waves, allowing an image of the structure inside the organism to be built (Mercuri et al., 2005). DXA is a measurement method using spectral imaging, which is based on the different attenuation coefficients of biological soft tissue and bone to X-rays. The internal tissue structure of the organism is thus imaged by appropriate weighting (Erlandson et al., 2016). However, above-mentioned techniques are expensive, complicated to operate, and unable to provide long-term continuous monitoring at bedside or home (Rubbieri et al., 2014). On another hand, physical muscle testing methods, such as hand grip strength (HGS) (Lauretani et al., 2003) and the 6-min walk test (6MWT) (Bennell et al., 2011) are commonly used to evaluate muscle function. However, to ensure the accuracy of the test, the observer needs to have a high professional level of competence (Cawthon, 2015). In addition, above-mentioned conventional methods require long preparation time which is unable to monitor the physiological response before and after *hybrid*EMS training in real-time.

In order to overcome the above-mentioned difficulties by conventional muscle evaluation methods, the electrical signal of human muscles should be detected since the electrical signal is directly reflected by the physiological response of muscle activity (Coggshall and Bekey, 1970). An electrical measurement is a promising approach to achieving real-time measurement to evaluate the physiological-induced conductive response of human muscle in *pre*-and *post*-*hybrid*EMS training. Previously, several fundamental electrical phenomena were investigated by electrical impedance measurement to characterize the oxidative stress in single skeletal muscle cells (Ferguson et al., 2021), by electromyography to access the hamstrings of healthy young men (de Caldas Honorato et al., 2021), and by the bio-impedance analysis (BIA) to measure overall muscle mass in the trunk and limbs (Di Vincenzo et al., 2021). However, conventional electrical impedance measurements are not able to provide adequate visualization of physiological-induced conductive response in specific muscle compartments in *pre*-and *post*-*hybrid*EMS training.

Electrical impedance tomography (EIT) first appeared in the 1980s as a safe, non-invasive, low-cost and real-time detection method that has matured into industrial applications and medical detections. Recently, the EIT is upgraded to frequency-difference EIT (*fd*EIT), which is performed to detect the physiological response areas of human calf muscles under single-segment EMS in *pre*-training, *post*-training, and relaxation (Sun et al., 2021a). *fd*EIT was performed to explore the differential tendency of physiological-induced conductive response in calf muscle compartments during voltage intensity change of EMS (*vic*-EMS) (Sun et al., 2021b). Since the *hybrid*EMS training are paid attention recently to enhancing the high-intensity exercise metabolic response in specific muscle compartments, we consider that tendency of physiological-induced conductive response changes over time after perform *hybrid*EMS, which indicates the effectiveness of *hybrid*EMS duration in specific muscle compartments.

Therefore, this study provides three objectives which are 1) to propose electrical impedance tomography (EIT) as a novel method to visualize the specific muscle compartments for *hybrid*EMS training, 2) to clarify the tendency of physiological-induced conductive response in the specific muscle compartments of human upper arm over time under three training modalities, and 3) to quantitatively evaluate the physiological-induced conductive response by three training modalities with conductance value *G* and extracellular water ratio *β* by bio-impedance analysis method.

## 2 Experiments and evaluation

### 2.1 Summary of *hybrid*EMS training

**FIGURE 1:**
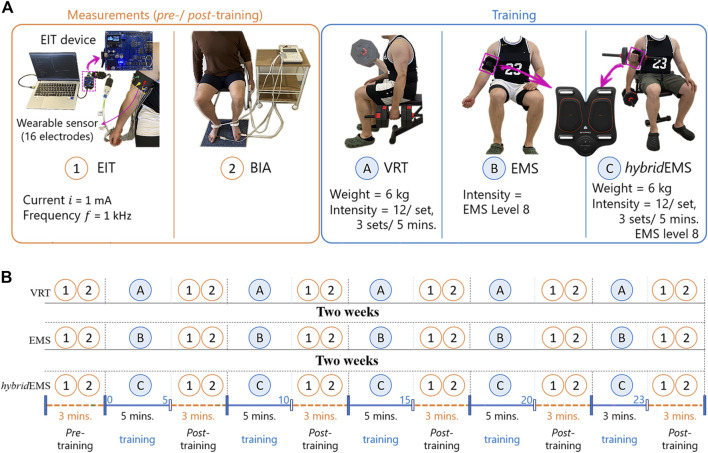
The experimental procedures. **(A)** the conducted measurements and training procedures and **(B)** experimental protocol.

Figure 1A shows the conducted measurements and training procedures. In the training procedure, three training modalities were performed in this study, namely voluntary resistance training (VRT), electrical muscle stimulation (EMS), and hybrid of VRT and EMS (*hybrid*EMS). In the VRT procedure, subjects were required to perform bicep curls using 6 kg dumbbell for 3 sets of 12 reps over 5 min under professional guidance. There is a 1-min break between each set. In the EMS procedure, subjects were required to perform continuous EMS stimulation of the biceps on right upper arm for 5 min. The commercial EMS equipment (SIXPAD Arm belt, Nagoya MTG Ltd., Japan) was employed in this study, which used a constant-controlled current (8 mA) but uses a different voltage value to adapt the training intensity. The stimulation cycle rule was 4 s stimulation with 4 s pause and a stimulation frequency of 20 Hz. After testing the EMS tolerance limit of each experimental subject, level 8 of EMS training intensity was selected from twenty training levels of EMS. Level 8 means that the EMS output has an average voltage of 22.99 V. In the *hybrid*EMS procedure, subjects were required to perform continuous EMS stimulation of the biceps on right upper arm with a training intensity of level 8 continuous stimulation for 5 min. Simultaneously, three sets of VRT procedure were performed on the subjects under professional guidance within 5 min, with a training intensity of 12 reps per set by using a 6 kg dumbbell.

### 2.2 Experimental method

In this study, eight healthy young men (age: 30 ± 7 years, height: 173 ± 10 cm, skeletal muscle mass: 34.8 ± 9.6 kg) volunteered for this study. All voluntary subjects gave informed consent. None of the subjects had any history of any musculoskeletal or neurological disorders. Figure 1B shows the experimental protocol consisting of three parts: *pre*-training, training, and *post*-training. In all experimental protocols, subjects were required a sitting position.

**FIGURE 2:**
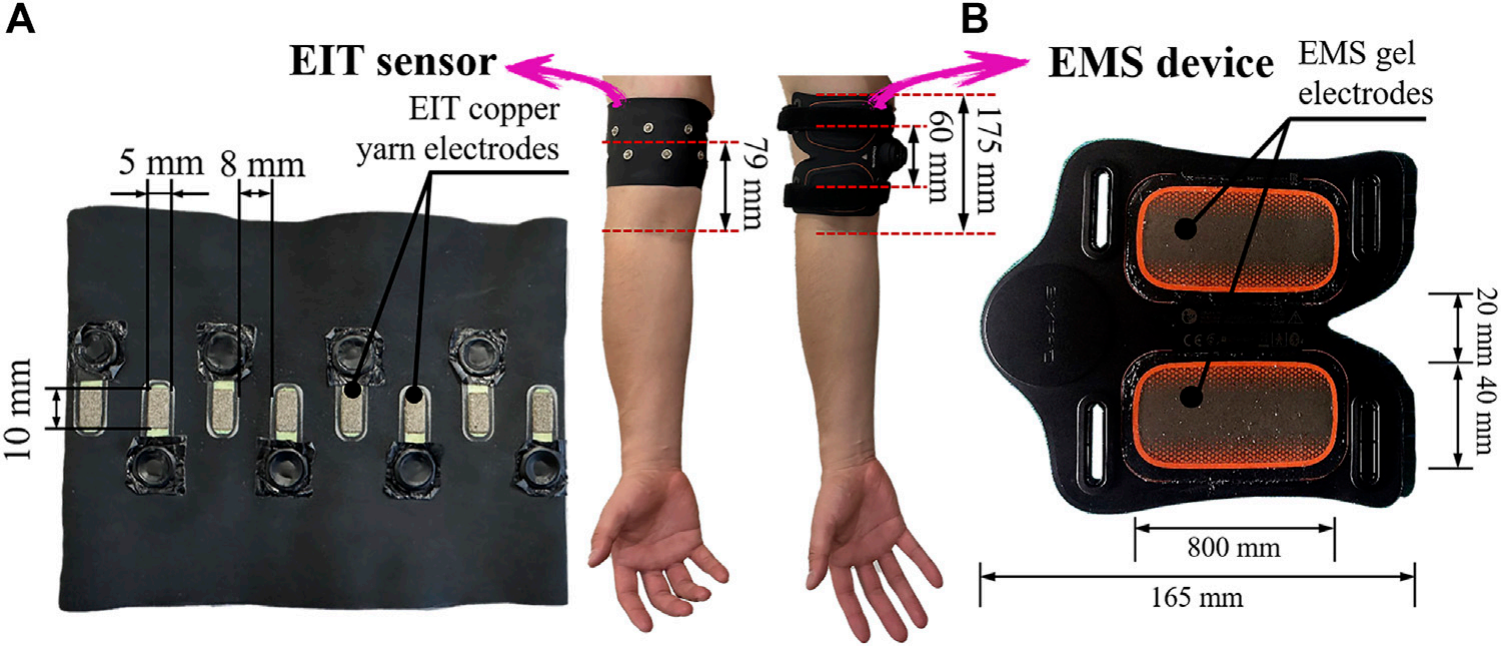
Electrode locations for **(A)** electrical impedance tomography (EIT) and **(B)** electrical muscle stimulation (EMS).

In the pre-training and post-training parts, firstly, EIT is conducted to reconstruct the conductivity distribution images **σ** ^*pre*^ in the right upper arm. Figure 1A shows the EIT muscle imaging system, which consists of four units: a portable EIT device (size: 85 mm × 70 mm; weight: 156 g), a wearable sensor consisting of 16 copper yarn electrodes, and a personal computer containing software for image reconstruction algorithms. In order to control the impedance measurement in each electrode, our portable EIT device has a sixteen-channel robust multiplexer (Sejati et al., 2022). The EIT electrodes were connected to the EIT device by a coaxial wire with a snap connector. Figure 2A shows the electrode location for electrical impedance tomography (EIT). In order to maintain the stability of the measurement results, ten consecutive measurements are taken in each measurement. The average boundary voltage **V** of the ten measurements were obtained which is used to reconstruct the conductivity distribution images **σ**. Secondly, BIA (InbodyS10, InBodyCo., Ltd., Korea) is able to measure the impedance value *Z*, the reactance value *Xc*, and the phase angle value *φ*, therefore, the conductance value *G* is obtained by:

$$R=\frac{X_{c}}{\arctan\varphi }$$ (1)

$$G=\frac{1}{R}$$ (2)

Also, the BIA method is conducted to measure the extracellular water ratio *β* = ECW/TBW, where ECW is extracellular water, TBW is total body water. Therefore, conductance value *G* and extracellular water ratio *β* are compared with the conductivity distribution changes measured from EIT method.

In the training part, in order to explore the tendency of physiological-induced conductive response changes by three training modalities. The same subjects were requested to visit the laboratory four times, 2 weeks between each visit. The first visit was to test and decide on the training load to be used for the experiment. The experiment decided to use a 6 kg dumbbell as the training load for resistance training and an output voltage of 22.99 V as the training load for EMS training, after testing the training tolerance limits of each subject. From the second visit, subjects were requested to perform three different training modalities, VRT, EMS, and *hybrid*EMS, as shown in Figure 2A. Since the EMS equipment system used in this experiment is set for a complete training session of 23 min, the training part of this study had a total training duration of 23 min, the training part in this study was divided into 5 periods which were 5, 10, 15, 20, and 23 min respectively.

### 2.3 Analysis and evaluation method of responsive muscle compartments

The image reconstruction algorithm to reconstruct **σ** from boundary voltage **V** use Gaussian-Newton method (Darma and Takei, 2021) expressed by

$$\sigma =J^{T}∆V-{\left(J^{T}J+\mu I\right)}^{-1}J^{T}∆V$$ (3)

where **J** is the Jacobian matrix, **J** ^*T*^ is the transpose of Jacobian matrix. *μ* is the hyperparameter, the best *μ* was chosen which error lied in the elbow of L-Curve (Braun et al., 2017). $\Delta V=\left[\Delta V_{1},\ldots ,\Delta V_{m},\ldots \Delta V_{M}\right]$ ϵ ℜ^*M*^ is the voltage difference between one measured voltage **V** ^*f*2^ at high frequency *f*_2_ injection current and another measured impedance **V** ^*f*1^ at low frequency *f*_1_ injection current in frequency difference EIT (Baidillah et al., 2017), which is expressed by

$$∆V_{m}=\frac{V_{m}^{f_{2}}-V_{m}^{f_{1}}}{V_{m}^{f_{1}}}$$ (4)

where, two frequencies are heuristically selected as *f*_1_ = 500 Hz and *f*_2_ = 1 kHz to obtain the best **σ** (Sun et al., 2021b).

**FIGURE 3:**
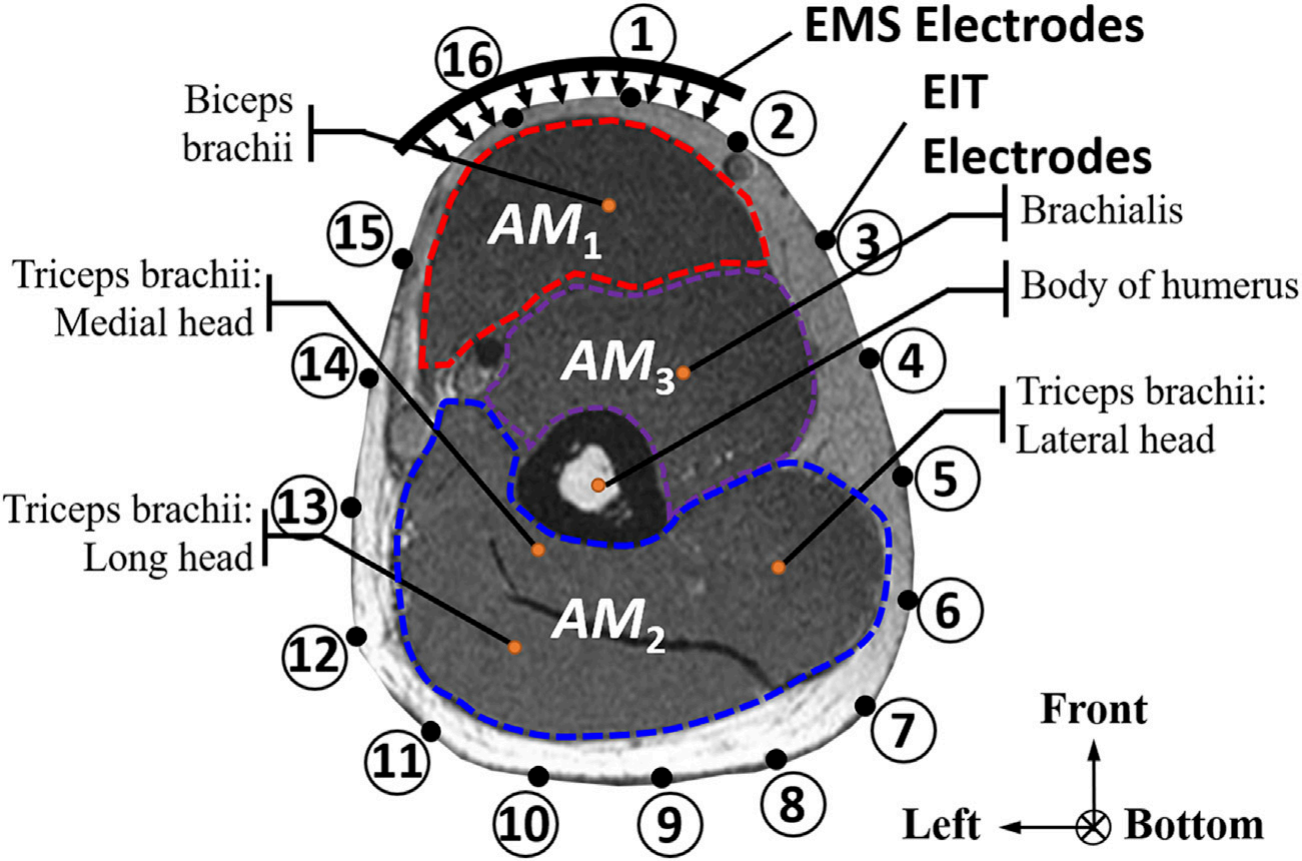
The structure of right arm muscle compartments from MRI images (33).

The conductivity distribution images **σ** show the upper arm muscle compartments of physiological-induced conductive response by three training modalities in post-training part, which are denoted as *AM*_1_, *AM*_2_ and *AM*_3_ compartments. Figure 3 shows the structure of responsive compartments. *AM*_1_ compartment composed of biceps brachii muscle, *AM*_2_ compartment composed of triceps brachii muscle, and *AM*_3_ compartment composed of brachialis muscle. According to our previous studies, conductivity images are sensitive to changes in muscle extracellular water (Sun et al., 2021a). In order to evaluate the effectiveness of three training modalities on muscle, the extracellular water ratio *β* was used to compare with the spatial-mean conductivity of specific muscle compartments.

### 2.4 Statistics

In order to clarify the tendency of physiological-induced conductive response in the specific muscle compartments of upper arm over time under three training modalities, a paired-samples *t*-test is performed in *AM*_1_, *AM*_2_ and *AM*_3_ compartments for the special-mean conductivity <**σ**>_*AM*1_, <**σ**>_*AM*2_ and <**σ**>_*AM*3_. A normality test is necessary prior to the analysis using paired-samples *t*-test. In this study, SPSS software (version 25.0) descriptive statistics function is used to examine the normal distribution of experimental data from EIT and BIA measurements. Table 1 shows the results of normality test. The null hypothesis is not rejected at the test level of *α* = 0.05, *p* > 0.05. Therefore, the experimental data from EIT and BIA measurements are considered to obey a normal distribution. The level of significance for paired-samples *t*-test was set at 0.05.

**TABLE 1:** The results of normal distribution examine.

				Resistance training				EMS training				*hybrid*EMS trianing				
				Kolomogorove-Smirnov		Shapiro-Wilk		Kolomogorove-Smirnov		Shapiro-Wilk		Kolomogorove-Smirnov			Shapiro-Wilk	
Condition		Items	*df*	Stats	Sig	Stats	Sig	Stats	Sig	Stats	Sig	Stats	Sig	Stats		Sig
Pre-training part		<**σ**>_*AM*1_	8	0.208	0.200	0.945	0.664	0.156	0.200	0.935	0.560	0.276	0.073	0.915		0.392
		<**σ**>_*AM*2_	8	0.200	0.200	0.917	0.403	0.251	0.146	0.916	0.398	0.187	0.200	0.962		0.830
		<**σ**>_*AM*3_	8	0.229	0.200	0.906	0.324	0.248	0.160	0.922	0.448	0.129	0.200	0.951		0.720
		*σ*	8	0.163	0.200	0.924	0.460	0.145	0.200	0.953	0.741	0.217	0.200	0.950		0.711
		*β*	8	0.240	0.198	0.816	0.052	0.214	0.200	0.889	0.230	0.187	0.200	0.920		0.434
Post-training part	5 min	<**σ**>_*AM*1_	8	0.214	0.200	0.856	0.109	0.149	0.200	0.935	0.562	0.174	0.200	0.958		0.794
		<**σ**>_*AM*2_	8	0.211	0.200	0.891	0.237	0.228	0.200	0.855	0.107	0.203	0.200	0.924		0.460
		<**σ**>_*AM*3_	8	0.179	0.200	0.913	0.372	0.355	0.064	0.763	0.051	0.143	0.200	0.983		0.977
		*σ*	8	0.162	0.200	0.943	0.644	0.176	0.200	0.933	0.540	0.208	0.200	0.961		0.816
		*β*	8	0.198	0.200	0.876	0.171	0.207	0.200	0.931	0.521	0.193	0.200	0.889		0.229
	10 min	<**σ**>_*AM*1_	8	0.208	0.200	0.892	0.244	0.229	0.200	0.834	0.065	0.217	0.200	0.896		0.263
		<**σ**>_*AM*2_	8	0.249	0.155	0.856	0.111	0.223	0.200	0.909	0.347	0.245	0.171	0.827		0.055
		<**σ**>_*AM*3_	8	0.267	0.097	0.853	0.102	0.237	0.200	0.844	0.082	0.193	0.200	0.899		0.281
		*σ*	8	0.177	0.200	0.940	0.607	0.166	0.200	0.916	0.398	0.153	0.200	0.976		0.939
		*β*	8	0.218	0.200	0.887	0.219	0.152	0.200	0.950	0.716	0.197	0.200	0.923		0.451
	15 min	<**σ**>_*AM*1_	8	0.291	0.054	0.880	0.189	0.182	0.200	0.936	0.577	0.289	0.052	0.887		0.221
		<**σ**>_*AM*2_	8	0.166	0.200	0.949	0.705	0.239	0.198	0.860	0.119	0.148	0.200	0.951		0.723
		<**σ**>_*AM*3_	8	0.231	0.200	0.826	0.054	0.184	0.200	0.965	0.857	0.146	0.200	0.957		0.778
		*σ*	8	0.231	0.200	0.906	0.324	0.153	0.200	0.924	0.462	0.148	0.200	0.970		0.899
		*β*	8	0.214	0.200	0.895	0.261	0.159	0.200	0.941	0.620	0.180	0.200	0.904		0.316
	20 min	<**σ**>_*AM*1_	8	0.261	0.115	0.816	0.052	0.171	0.200	0.977	0.946	0.188	0.200	0.935		0.561
		<**σ**>_*AM*2_	8	0.216	0.200	0.948	0.687	0.207	0.200	0.952	0.727	0.246	0.169	0.875		0.167
		<**σ**>_*AM*3_	8	0.199	0.200	0.943	0.637	0.242	0.186	0.915	0.393	0.248	0.158	0.852		0.100
		*σ*	8	0.227	0.200	0.886	0.217	0.158	0.200	0.913	0.373	0.157	0.200	0.947		0.682
		*β*	8	0.193	0.200	0.928	0.502	0.170	0.200	0.943	0.641	0.145	0.200	0.933		0.548
	23 min	<**σ**>_*AM*1_	8	0.371	0.052	0.642	0.051	0.259	0.121	0.844	0.083	0.211	0.200	0.917		0.405
		<**σ**>_*AM*2_	8	0.334	0.059	0.718	0.052	0.210	0.200	0.900	0.287	0.198	0.200	0.949		0.703
		<**σ**>_*AM*3_	8	0.192	0.200	0.889	0.230	0.167	0.200	0.938	0.592	0.254	0.138	0.854		0.105
		*σ*	8	0.238	0.200	0.898	0.279	0.168	0.200	0.902	0.300	0.148	0.200	0.956		0.776
		*β*	8	0.184	0.200	0.946	0.667	0.218	0.200	0.931	0.528	0.157	0.200	0.937		0.580

## 3 Experimental results

### 3.1 Reconstructed images

Table 2 shows the conductivity distribution images **σ** of eight subjects’ right upper arm in *AM*_1_, *AM*_2_, and *AM*_3_ under three training modalities obtained by Eq. 3. From the images **σ**, the conductivity distribution between pre-training and post-training under three training modalities, namely voluntary resistance training (VRT), electrical muscle stimulation (EMS), and hybrid of VRT and EMS (*hybrid*EMS), are clearly detected, which shows the tendency of physiological-induced conductive response are increased in all three training modalities with increasing training time. According to **σ**, the physiological-induced conductive response of *AM*_1_ compartment is significantly affected by all three training modalities. In contrast, the physiological-induced conductive response of *AM*_3_ compartment is moderately affected. And the physiological-induced conductive response of *AM*_2_ compartment is minimally affected by training.

**TABLE 2:** Conductivity distribution images **σ** in pre-training and post-training parts reconstructed by EIT under three training modalities.

Subject		σ^*pre*^	σ^5mins^	σ^10minss^	σ^15mins^	σ^20mins^	σ^23mins^	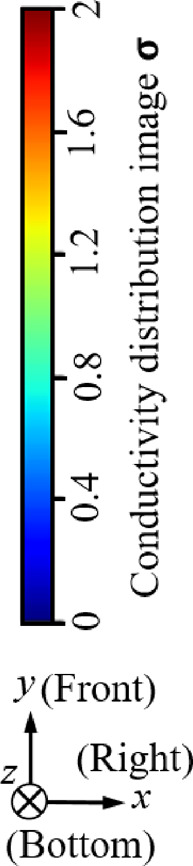
1	VRT	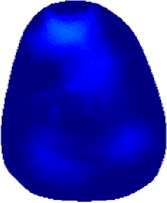	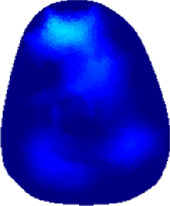	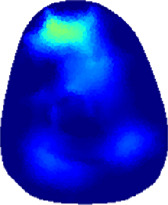	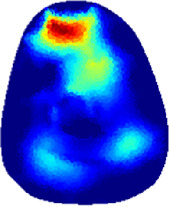	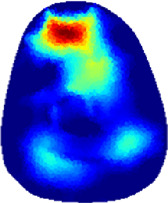	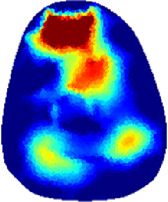	
	EMS	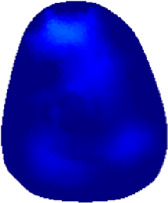	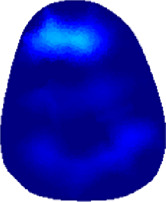	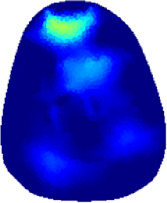	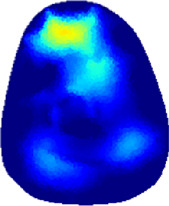	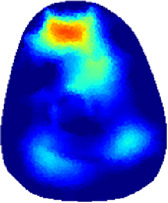	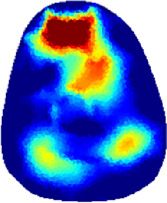	
	*hybrid*EMS	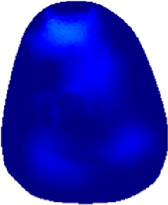	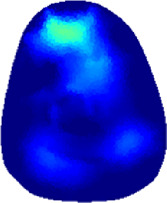	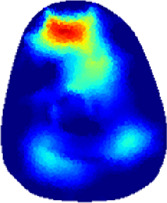	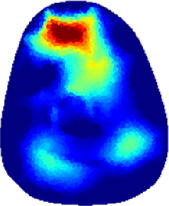	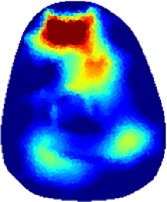	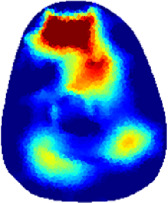	
2	VRT	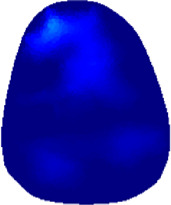	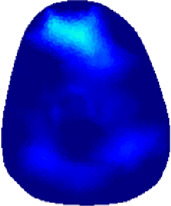	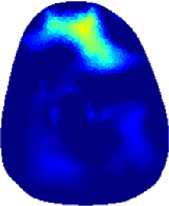	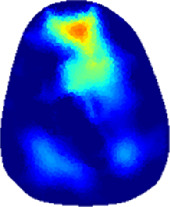	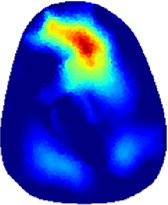	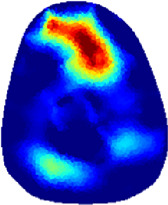	
	EMS	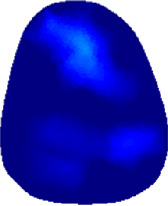	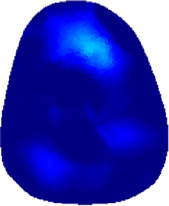	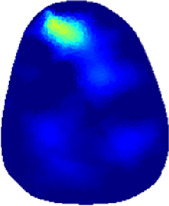	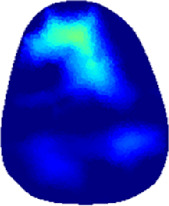	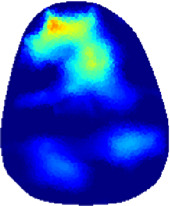	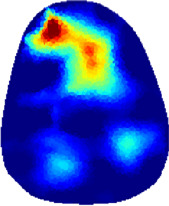	
	*hybrid*EMS	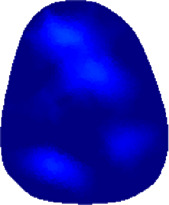	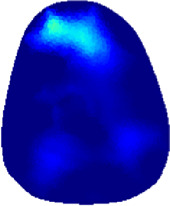	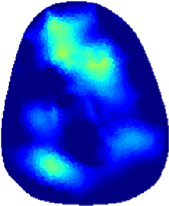	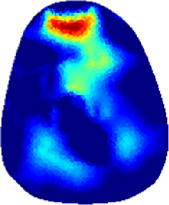	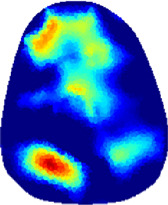	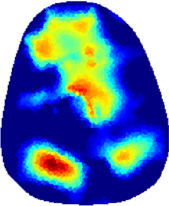	
3	VRT	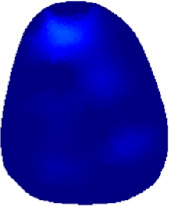	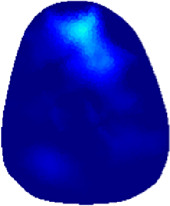	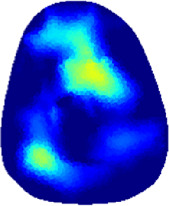	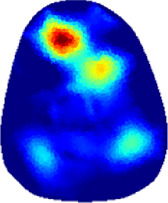	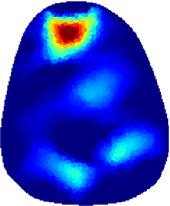	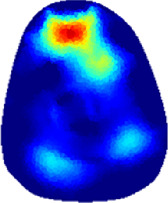	
	EMS	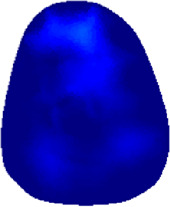	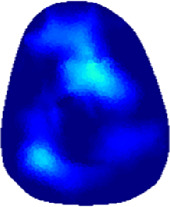	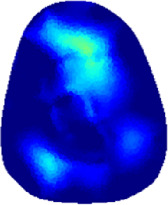	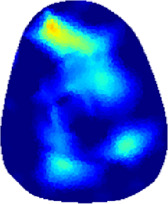	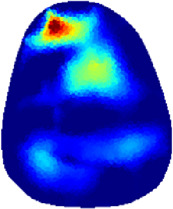	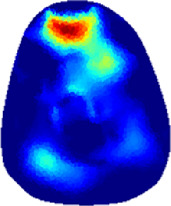	
	*hybrid*EMS	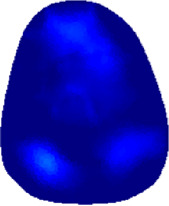	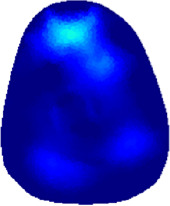	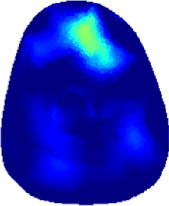	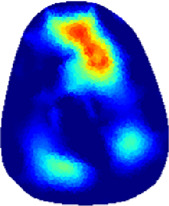	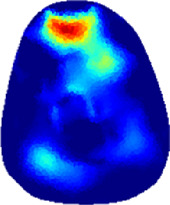	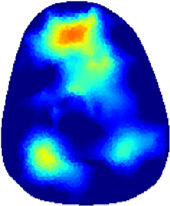	
4	VRT	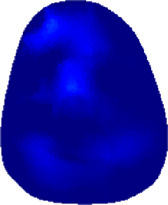	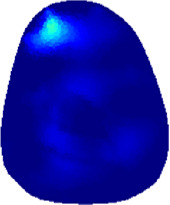	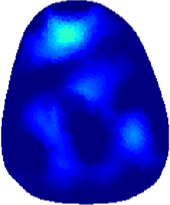	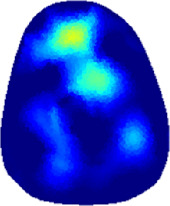	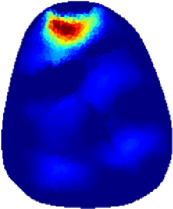	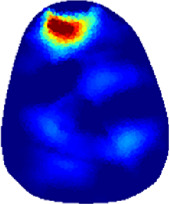	
	EMS	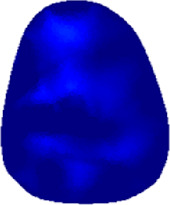	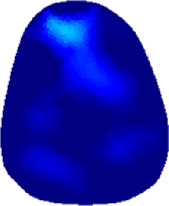	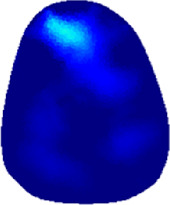	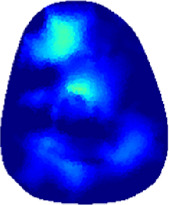	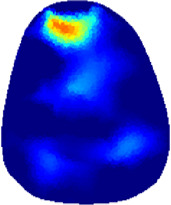	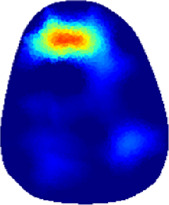	
	*hybrid*EMS	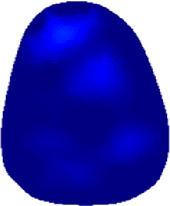	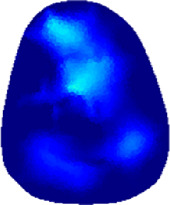	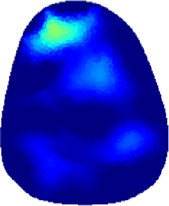	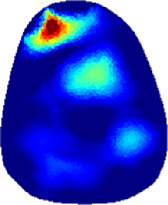	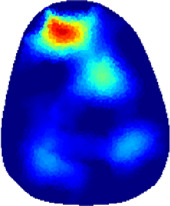	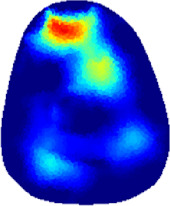	
5	VRT	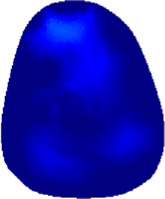	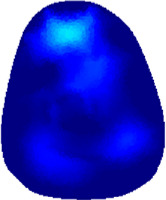	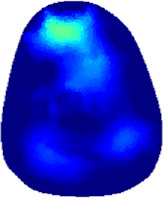	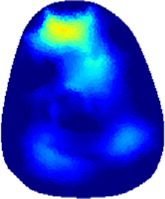	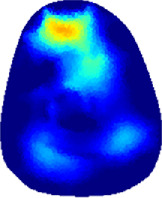	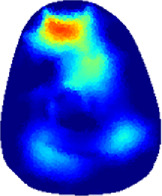	
	EMS	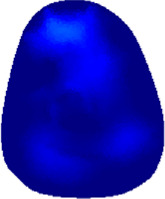	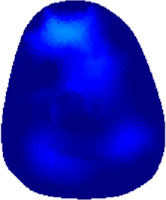	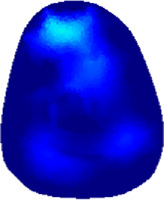	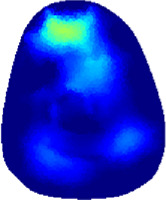	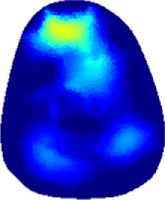	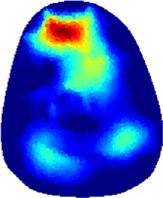	
	*hybrid*EMS	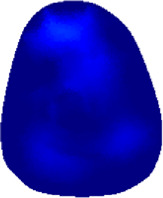	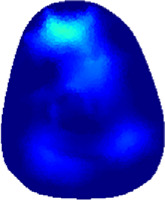	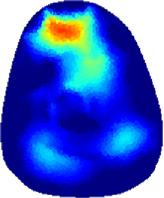	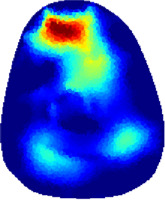	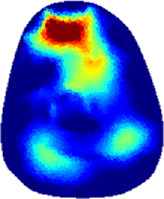	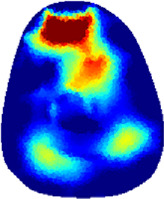	
6	VRT	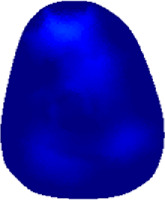	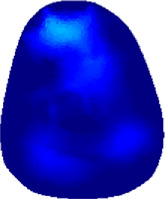	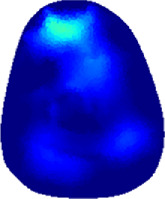	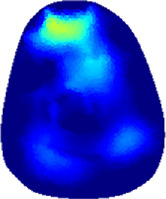	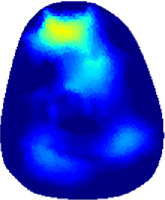	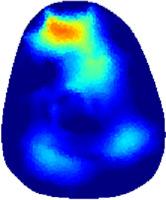	
	EMS	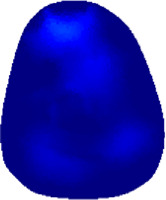	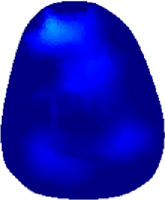	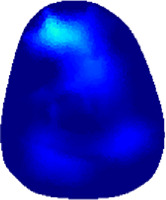	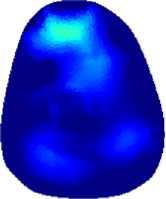	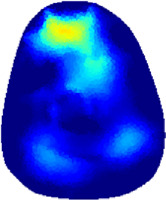	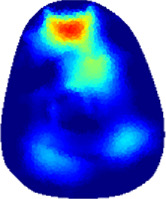	
	*hybrid*EMS	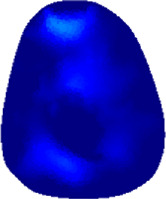	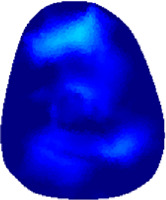	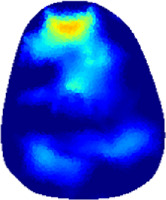	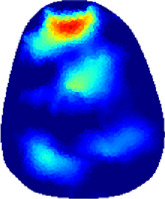	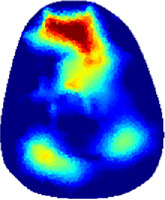	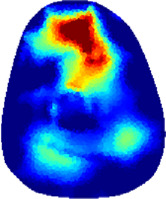	
7	VRT	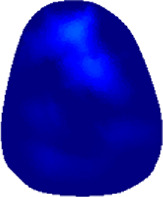	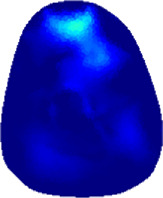	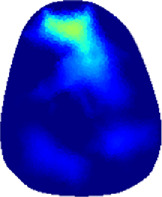	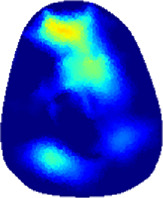	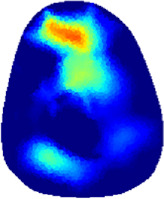	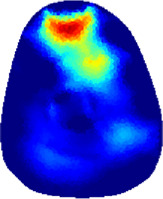	
	EMS	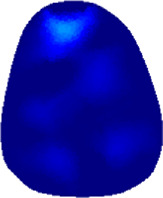	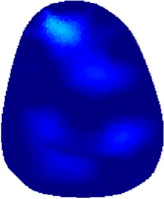	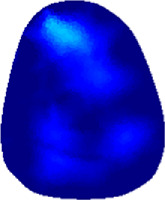	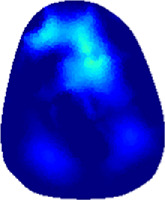	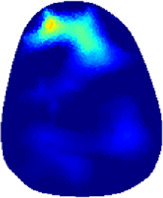	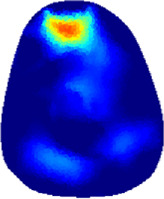	
	*hybrid*EMS	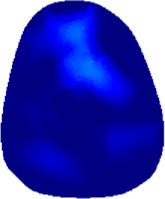	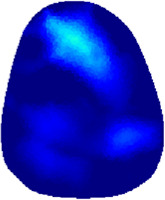	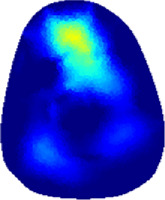	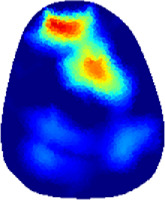	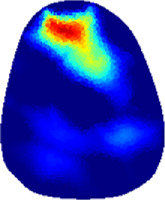	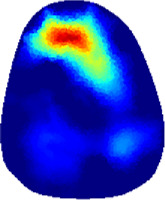	
8	VRT	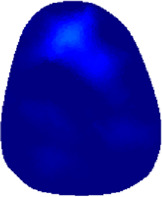	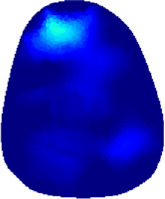	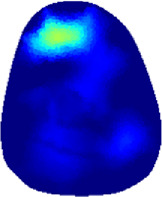	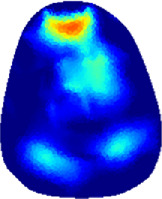	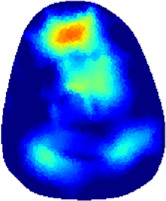	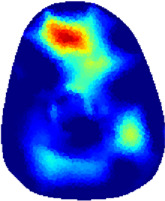	
	EMS	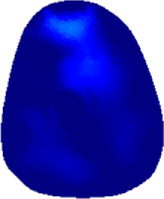	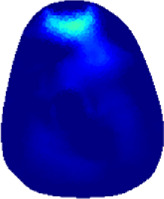	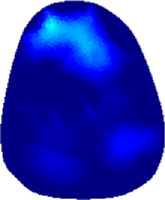	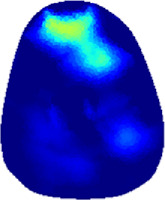	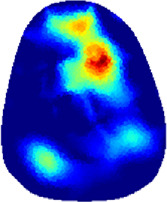	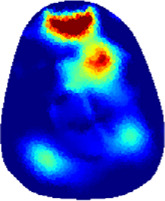	
	*hybrid*EMS	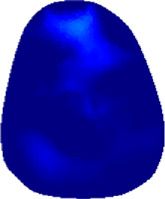	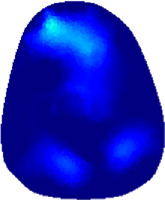	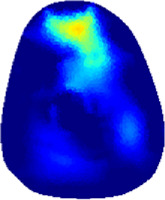	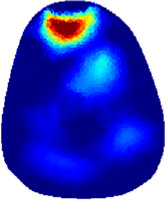	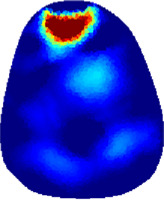	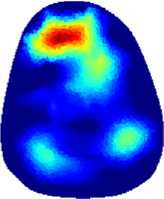	

From the images **σ** ^5mins^, the conductivity distribution of the *AM*_1_ compartment is significantly changed between *pre*- and *post*-*hybrid*EMS training compared to VRT and EMS, which indicates a faster effect of *hybrid*EMS on the upper arm muscles compared to the other two training modalities. Furthermore, the images **σ** ^23mins^ show that *hybrid*EMS is more effective in stimulating deeper muscle compartment (*AM*_3_) than VRT and EMS. The *hybrid*EMS has the greatest effect on physiological-induced conductive response for the same training time.

### 3.2 Paried-samples *t*-test results

**FIGURE 4:**
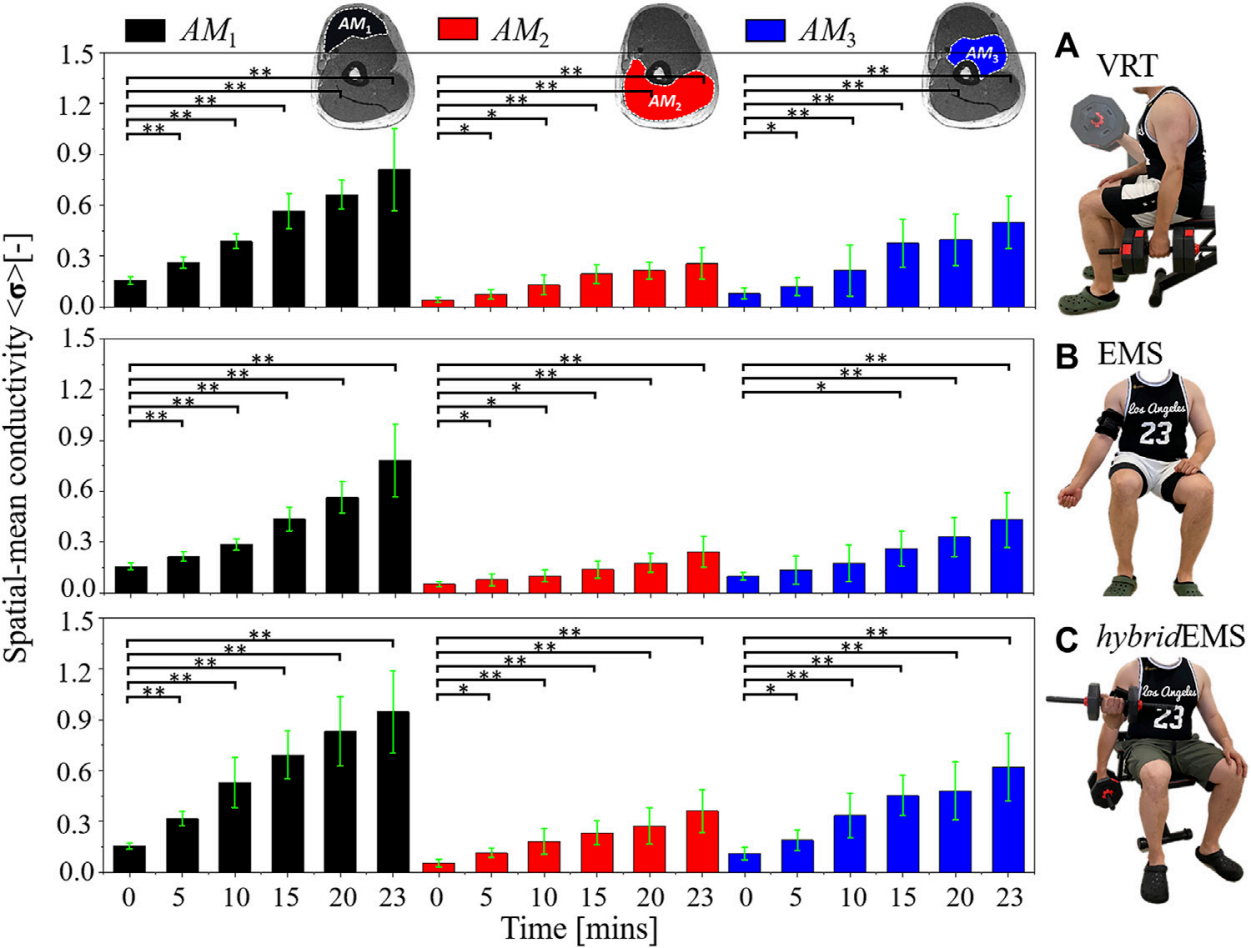
The paired-samples *t*-test results of spatial-mean conductivity <**σ**>_*AM*1,*AM*2,*AM*3_ between pre-training and post-training parts under **(A)** voluntary resistance training (VRT), **(B)** electrical muscle stimulation (EMS), and **(C)** hybrid of resistance training and EMS (*hybrid*EMS). *p < 0.05, **p < 0.01.

In order to quantitatively evaluate the physiological-induced conductive response of specific muscle compartments in right upper arm under three training modalities, the spatial-mean conductivity <**σ**>_*AM*1,*AM*2,*AM*3_ were obtained by a dedicated Python script which analysed the conductivity distribution images **σ**. Figure 4 shows the paired-samples *t*-test results of spatial-mean conductivity <**σ**>_*AM*1,*AM*2,*AM*3_ between pre-training and post-training parts under VRT, EMS, and *hybrid*EMS.

Under VRT, the spatial-mean conductivity increased from <**σ** ^*pre*^ > _*AM*1_ = 0.154 to <**σ** ^5mins^ > _*AM*1_ = 0.262, <**σ** ^10mins^ > _*AM*1_ = 0.386, <**σ** ^15mins^ > _*AM*1_ = 0.565, <**σ** ^20mins^ > _*AM*1_ = 0.662, <**σ** ^23mins^ > _*AM*1_ = 0.810 in *AM*_1_ muscle compartment (*n* = 8, *p* < 0.001); the spatial-mean conductivity increased from <**σ** ^*pre*^ > _*AM*2_ = 0.040 to <**σ** ^5mins^ > _*AM*2_ = 0.072, <**σ** ^10mins^ > _*AM*2_ = 0.128, <**σ** ^15mins^ > _*AM*2_ = 0.193, <**σ** ^20mins^ > _*AM*2_ = 0.213, <**σ** ^23mins^ > _*AM*2_ = 0.254 in *AM*_2_ muscle compartment (*n* = 8, *p* < 0.05); the spatial-mean conductivity increased from <**σ** ^*pre*^ > _*AM*3_ = 0.078 to <**σ** ^5mins^ > _*AM*3_ = 0.119, <**σ** ^10mins^ > _*AM*3_ = 0.213, <**σ** ^15mins^ > _*AM*3_ = 0.374, <**σ** ^20mins^ > _*AM*3_ = 0.394, <**σ** ^23mins^ > _*AM*3_ = 0.497 in *AM*_3_ muscle compartment (*n* = 8, *p* < 0.05).

Under EMS, the spatial-mean conductivity increased from <**σ** ^*pre*^ > _*AM*1_ = 0.155 to <**σ** ^5mins^ > _*AM*1_ = 0.214, <**σ** ^10mins^ > _*AM*1_ = 0.285, <**σ** ^15mins^ > _*AM*1_ = 0.436, <**σ** ^20mins^ > _*AM*1_ = 0.563, <**σ** ^23mins^ > _*AM*1_ = 0.782 in *AM*_1_ muscle compartment (*n* = 8, *p* < 0.001); the spatial-mean conductivity increased from <**σ** ^*pre*^ > _*AM*2_ = 0.052 to <**σ** ^5mins^ > _*AM*2_ = 0.076, <**σ** ^10mins^ > _*AM*2_ = 0.100, <**σ** ^15mins^ > _*AM*2_ = 0.137, <**σ** ^20mins^ > _*AM*2_ = 0.177, <**σ** ^23mins^ > _*AM*2_ = 0.242 in *AM*_2_ muscle compartment (*n* = 8, *p* < 0.05); the spatial-mean conductivity increased from <**σ** ^*pre*^ > _*AM*3_ = 0.096 to <**σ** ^5mins^ > _*AM*3_ = 0.133 (*n* = 8, *p* > 0.05), <**σ** ^10mins^ > _*AM*3_ = 0.176 (*n* = 8, *p* > 0.05), <**σ** ^15mins^ > _*AM*3_ = 0.261 (*n* = 8, *p* < 0.05), <**σ** ^20mins^ > _*AM*3_ = 0.329 (*n* = 8, *p* < 0.001), <**σ** ^23mins^ > _*AM*3_ = 0.431 (*n* = 8, *p* < 0.001) in *AM*_3_ muscle compartment.

Under *hybrid*EMS, the spatial-mean conductivity increased from <**σ** ^*pre*^ > _*AM*1_ = 0.153 to <**σ** ^5mins^ > _*AM*1_ = 0.316, <**σ** ^10mins^ > _*AM*1_ = 0.529, <**σ** ^15mins^ > _*AM*1_ = 0.691, <**σ** ^20mins^ > _*AM*1_ = 0.830, <**σ** ^23mins^ > _*AM*1_ = 0.947 in *AM*_1_ muscle compartment (*n* = 8, *p* < 0.001); the spatial-mean conductivity increased from <**σ** ^*pre*^ > _*AM*2_ = 0.053 to <**σ** ^5mins^ > _*AM*2_ = 0.112, <**σ** ^10mins^ > _*AM*2_ = 0.181, <**σ** ^15mins^ > _*AM*2_ = 0.231, <**σ** ^20mins^ > _*AM*2_ = 0.273, <**σ** ^23mins^ > _*AM*2_ = 0.359 in *AM*_2_ muscle compartment (*n* = 8, *p* < 0.05); the spatial-mean conductivity increased from <**σ** ^*pre*^ > _*AM*3_ = 0.109 to <**σ** ^5mins^ > _*AM*3_ = 0.188, <**σ** ^10mins^ > _*AM*3_ = 0.335, <**σ** ^15mins^ > _*AM*3_ = 0.452, <**σ** ^20mins^ > _*AM*3_ = 0.478, <**σ** ^23mins^ > _*AM*3_ = 0.621 in *AM*_3_ muscle compartment (*n* = 8, *p* < 0.05). Therefore, the quantitative results also highlight that *hybrid*EMS has the greatest effect on physiological-induced conductive response for the same training time. In addition, EMS has a slower effect on deep muscle compartment (*AM*_3_) compared to VRT and *hybrid*EMS, with a significant difference between <**σ** ^*pre*^ > _*AM*3_ and <**σ** ^15mins^ > _*AM*3_ after 15 min of training.

**FIGURE 5:**
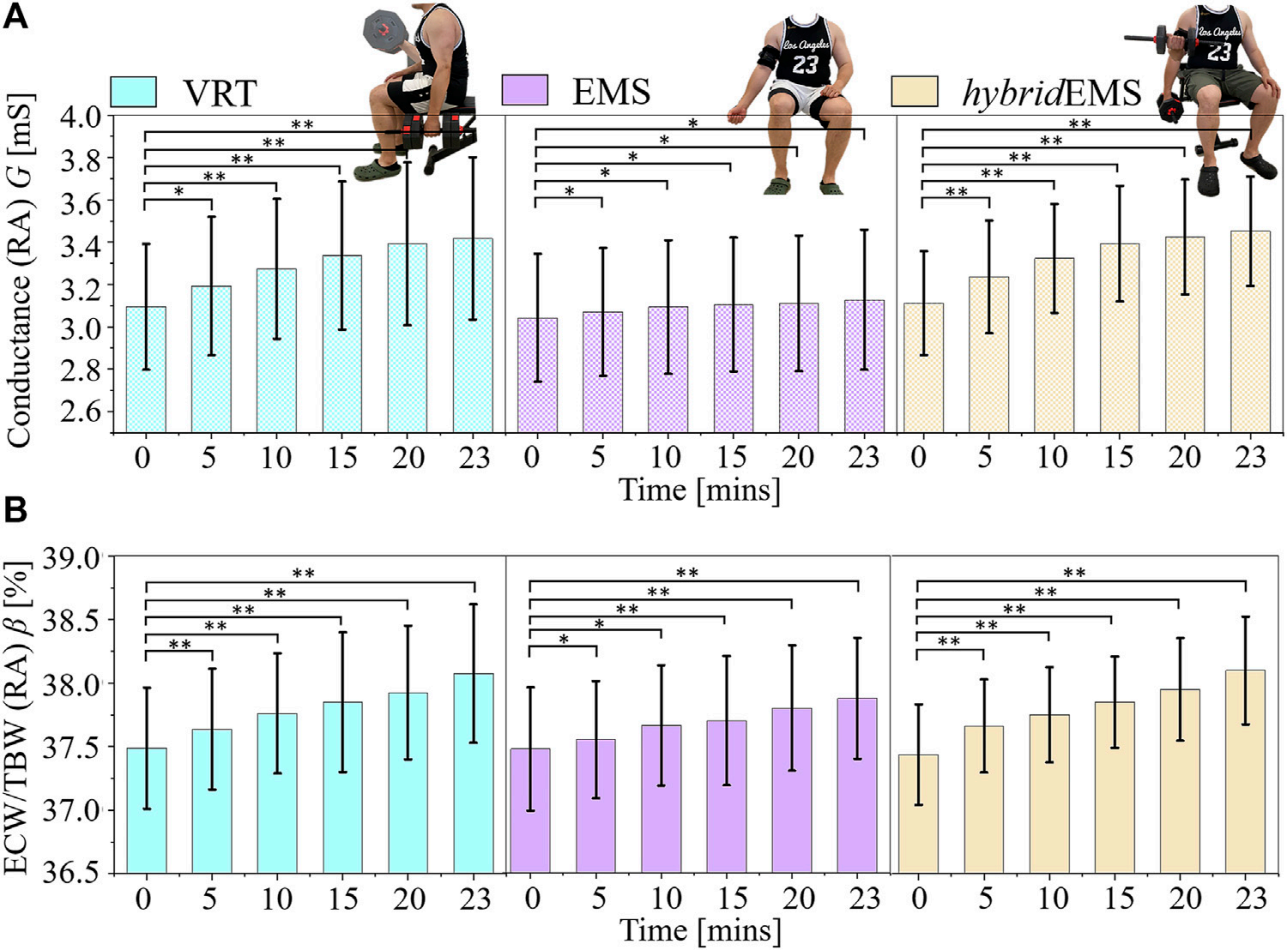
The paired-samples *t*-test results of **(A)** conductance value *G* and **(B)** extracellular water ratio *β* between pre-training and post-training parts under voluntary resistance training (VRT), electrical muscle stimulation (EMS), and hybrid of VRT and EMS (*hybrid*EMS). *p < 0.05, **p < 0.01.

Figure 5A shows the paired-samples *t*-test results of conductance value *G* between pre-training and post-training parts under VRT, EMS, and *hybrid*EMS. Under VRT, the conductance value increased from *G* ^*pre*^ = 3.09 mS to *G* ^5mins^ = 3.19 mS, *G* ^10mins^ = 3.27 mS, *G* ^15mins^ = 3.33 mS, *G* ^20mins^ = 3.39 mS, *G* ^23mins^ = 3.41 mS (*n* = 8, *p* < 0.05). Under EMS, the conductance value increased from *G* ^*pre*^ = 3.04 mS to *G* ^5mins^ = 3.06 mS, *G* ^10mins^ = 3.09 mS, *G* ^15mins^ = 3.10 mS, *G* ^20mins^ = 3.11 mS, *G* ^23mins^ = 3.12 mS (*n* = 8, *p* < 0.05). Under *hybrid*EMS, the conductance value increased from *G* ^*pre*^ = 3.11 mS to *G* ^5mins^ = 3.23 mS, *G* ^10mins^ = 3.32 mS, *G* ^15mins^ = 3.39 mS, *G* ^20mins^ = 3.42 mS, *G* ^23mins^ = 3.45 mS (*n* = 8, *p* < 0.001).

Figure 5B shows the paired-samples *t*-test results of extracellular water ratio *β* between pre-training and post-training parts under VRT, EMS, and *hybrid*EMS. Under VRT, the conductance value increased from *β* ^*pre*^ = 37.48% to *β* ^5mins^ = 37.63%, *β* ^10mins^ = 37.76%, *β* ^15mins^ = 37.85%, *β* ^20mins^ = 37.92%, *β* ^23mins^ = 38.07% (*n* = 8, *p* < 0.001). Under EMS, the conductance value increased from *β* ^*pre*^ = 37.47% to *β* ^5mins^ = 37.55%, *β* ^10mins^ = 37.66%, *β* ^15mins^ = 37.70%, *β* ^20mins^ = 37.80%, *β* ^23mins^ = 37.87% (*n* = 8, *p* < 0.05). Under *hybrid*EMS, the conductance value increased from *β* ^*pre*^ = 37.43% to *β* ^5mins^ = 37.66%, *β* ^10mins^ = 37.75%, *β* ^15mins^ = 37.85%, *β* ^20mins^ = 37.95%, *β* ^23mins^ = 38.10% (*n* = 8, *p* < 0.001). Therefore, the tendency of <**σ**>_*AM*1,*AM*2,*AM*3_ is the same as the tendency of *G* and *β* under VRT, EMS and *hybrid*EMS.

## 4 Discussion

### 4.1 Physiological-induced conductive response in specific muscle compartments

Physiological-induced conductive response in specific muscle compartments of right upper arm under three training modalities are discussed from the viewpoint of sports anatomy. Firstly, two muscle compartments are located below the electrodes of electrical muscle stimulation (EMS), which are called *AM*_1_ compartment composed of biceps brachii muscle and *AM*_3_ compartment composed of brachialis muscle. From a previous study, the biceps brachii muscle and brachialis muscle are recognized to be mainly involved in the type II (fast-twitch) muscle fibres (Klein et al., 2003), which are recruited more quickly under voluntary resistance training (VRT), electrical muscle stimulation (EMS), and hybrid of VRT and EMS (*hybrid*EMS). However, the brachialis muscle located behind the biceps brachii muscle (farther from the EMS electrodes) is not able to elicit a physiological-inducted conductive response by EMS in a short time. According to a recent study, EMS provides undifferentiated stimulation of only the most superficial muscle fibres under the electrodes (Gregory and Bickel, 2005). Therefore, the spatial-mean conductivity has a significant difference between <**σ** ^*pre*^ > _*AM*3_ and <**σ** ^15mins^ > _*AM*3_ after 15 min of training under EMS, as shown in Figure 4B.

**FIGURE 6:**
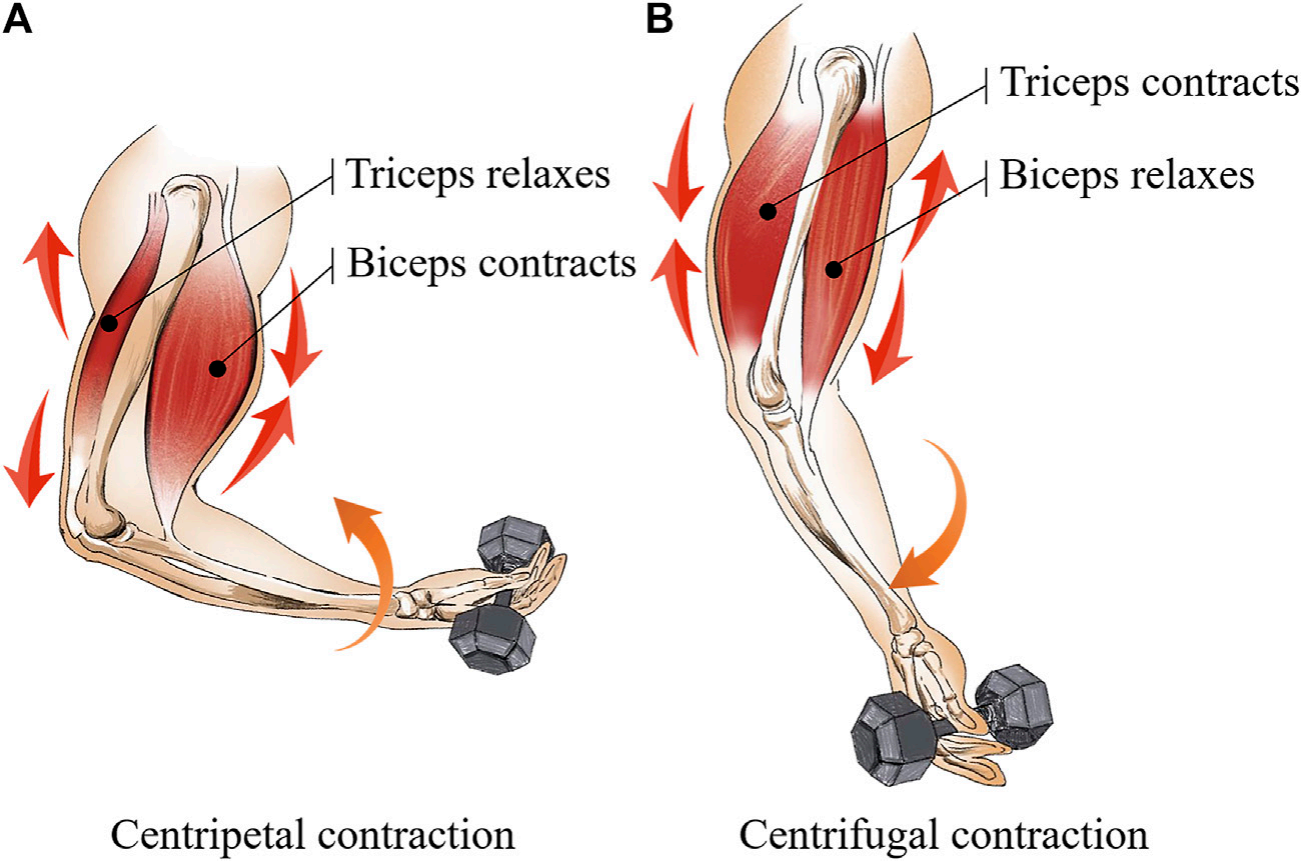
Two processes are included in biceps curl movement **(A)** the centripetal contraction and **(B)** the centrifugal contraction.

Secondly, the biceps curl movement consists of two processes, namely the centripetal contraction and the centrifugal contraction (Kenney et al., 2021). Figure 6A shows the centripetal contraction of the biceps is defined as the movement of the elbow joint from extension to flexion, in which the target muscle outputs a force larger than the loading weight. Figure 6B shows the centrifugal contraction of the biceps is defined as the movement of the elbow joint from flexion to extension, in which the target muscle outputs a force less than the loading weight. Moreover, since the biceps and triceps are mutually antagonistic muscles (Gilhodes et al., 1986), the centrifugal process of the biceps is the centripetal process of the triceps. However, due to the fact that the triceps are not trained against the weight during the biceps curl. *AM*_2_ compartment, which is recognized as the position of the triceps brachii muscle, has a less physiological-induced conductive response compared to *AM*_1_ and *AM*_3_ compartments under VRT. Similarly, EMS is applied above the biceps, the EMS stimulation current does not induce contraction of the triceps. The physiological-induced conductive response of *AM*_3_ compartment after 15 min resulted from the triceps working in concert with the biceps to complete the arm bending movement without loading weight, therefore, the spatial-mean conductivity <**σ**>_*AM*3_ under EMS is less than VRT at the same training time.

Thirdly, synthesizing the above-mentioned reasons from a sports anatomy viewpoint, the physiological-induced conductive response of *hybrid*EMS compared to VRT and EMS for specific muscle compartments is discussed. Due to the ability of EMS to activate muscle fibres that would not normally be used in the daily activities (Gregory and Bickel, 2005). Thus, the spatial-mean conductivity <**σ**>_*AM*1,*AM*3_ are significantly larger under *hybrid*EMS compared to VRT and EMS at the same training time. In addition, under *hybrid*EMS, the biceps muscle is loaded by EMS during centrifugal contraction, which requires the triceps muscle to work against the additional load generated by EMS. Therefore, the spatial-mean conductivity <**σ**>_*AM*2_ is significantly larger under *hybrid*EMS compared to VRT and EMS.

### 4.2 Individual differences on physiological-induced conductive response

**FIGURE 7:**
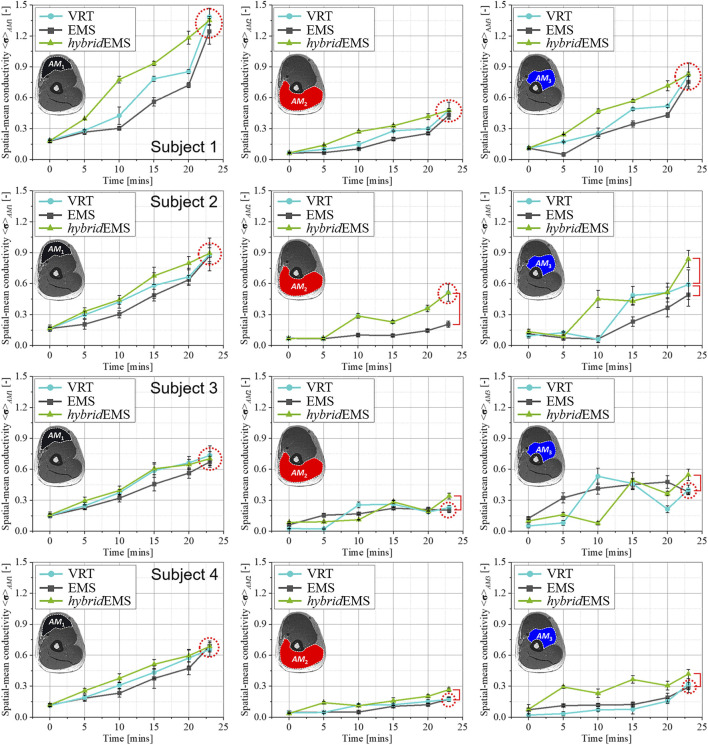
The relationship between spatial-mean conductivity <**σ**>_*AM*1,*AM*2,*AM*3_ and training time in *AM*_1_, *AM*_2_, and *AM*_3_ compartments under VRT, EMS, and hybrid EMS of upper arm in *std*VRT group.

**FIGURE 8:**
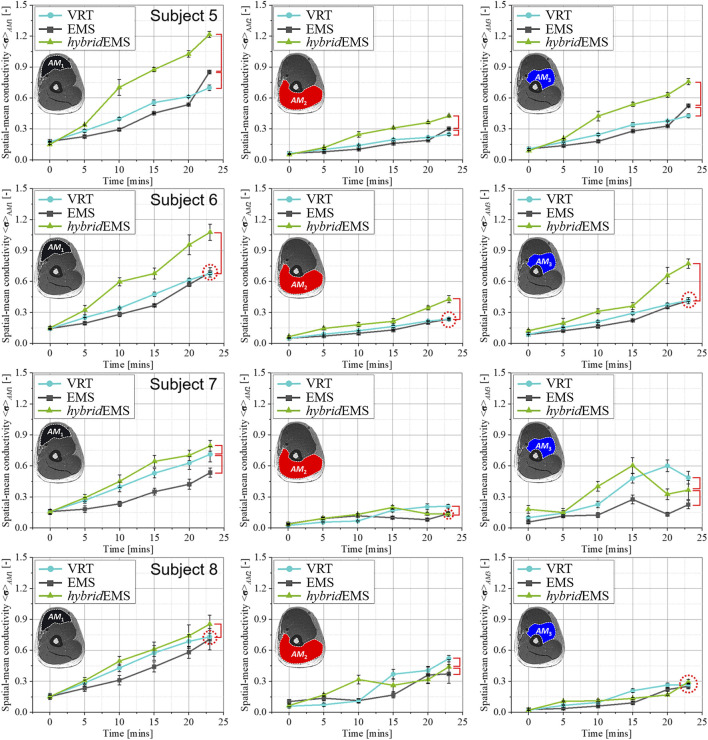
The relationship between spatial-mean conductivity <**σ**>_*AM*1,*AM*2,*AM*3_ and training time in *AM*_1_, *AM*_2_, and *AM*_3_ compartments under VRT, EMS, and hybrid EMS of upper arm in *un*VRT group.

Figures 7, 8 show the relationship between spatial-mean conductivity <**σ**>_*AM*1,*AM*2,*AM*3_ and training time in *AM*_1_, *AM*_2_, and *AM*_3_ compartments under VRT, EMS, and *hybrid*EMS of eight subjects’ right upper arm. Individual differences on physiological-induced conductive response of the upper arm muscles are significant between the three training modalities. Across all subjects, the tendency of spatial-mean conductivity <**σ**>_*AM*1_ in *AM*_1_ compartment shows a maximum increasing slope under *hybrid*EMS over the time, which implies an efficient elicitation of physiological-induced response by *hybrid*EMS compared to VRT and EMS for all subjects. Subjects are categorised into two groups, namely people who are able to perform standard VRT (*std*VRT) group and people who are not able to perform standard VRT (*un*VRT) group. In *std*VRT group, the spatial-mean conductivity <**σ** ^23mins^ > _*AM*1,*AM*2,*AM*3_ under three training modalities showed a minor difference in values, as shown in Figure 7. In contrast, the spatial-mean conductivity <**σ** ^23mins^ > _*AM*1,*AM*2,*AM*3_ under three training modalities showed a significant difference in values, as shown in Figure 8. Therefore, in order to achieve the same physiological-induced conductive response, *hybrid*EMS takes much shorter time compared to VRT and EMS. *hybrid*EMS is recommended for people who are unable to perform standard voluntary resistance training, because *hybrid*EMS has a better training effectiveness over the same training time.

## 5 Conclusion

The present study reveals that the physiological-induced conductive response of specific muscle compartments in human upper arm under three training modalities have been detected by electrical impedance tomography (EIT). The key findings of this study are as follows.

1) Based on the reconstructed images, the conductivity distribution between pre-training and post-trarining parts under three training modalities are clearly detected by EIT, which shows the tendency of physiological-induced conductive response are increased in all three training modalities with increasing training time.
2) In the post-training part, the spatial-mean conductivity <**σ**>_*AM*1,*AM*2,*AM*3_ increases as the conductance value *G* and extracellular water ratio *β* of right arm by bio-impedance analysis (BIA) method increase.
3) The paired samples *t*-test results of this investigation demonstrate that *hybrid*EMS has the greatest effect on physiological induced conductive response for the same training time. EMS has a slower effect on deep muscle compartment, *AM*_3_ is recognized as brachialis muscle, compared to VRT and *hybrid*EMS, with a significant difference between <**σ** ^*pre*^ > _*AM*3_ and <**σ** ^15mins^ > _*AM*3_ after 15 min of training.

## Data Availability

The raw data supporting the conclusion of this article will be made available by the authors, without undue reservation.
